# Terminological and Epistemological Issues in Current Memory Research

**DOI:** 10.3389/fnmol.2019.00336

**Published:** 2020-01-22

**Authors:** Oliver Hardt, Wayne S. Sossin

**Affiliations:** ^1^Department of Psychology, McGill University, Montreal, QC, Canada; ^2^The Simons Initiative for the Developing Brain and The Patrick Wild Centre, The University of Edinburgh, Edinburgh, United Kingdom; ^3^The Montreal Neurological Institute and Hospital, McGill University, Montreal, QC, Canada

**Keywords:** engram, consolidation, synapse, memory, system consolidation, reconsolidation, forgetting

## Abstract

A number of observations in recent years demonstrates that across all levels of organization, memory is inherently fluid. On the cognitive-behavioral level, the innocent act of remembering can irrevocably alter the contents of established long-term memories, while the content of dormant long-term memories that is deemed irrelevant, superfluous, or limiting may be pragmatically erased or suppressed. On the cellular level, the proteins implementing the molecular alterations underpinning memories are in a constant state of flux, with proteins being turned over, translocated, reconfigured, substituted, and replaced. Yet, the general perception of memory, and the words used to describe it, suggest a static system characterized by the goal of preserving records of past experiences with high fidelity, in contrast to the reality of an inherently adaptive system purposed to enable survival in a changing world with a pragmatic disregard for the fate of acquired memories. Here, we examine present memory terminology and how it corresponds to our actual understanding of the molecules, cells, and systems underlying memory. We will identify where terms lead us astray and line out possible ways to reform memory nomenclature to better fit the true nature of memory as we begin to know it.

“*Philosophy is a battle against the bewitchment of our intelligence by means of our language.*”

(Wittgenstein 1973, Philosophical Investigations)

Psychologists use the word “*memory*” in broad reference to mental states that are qualitatively similar to the experience of seeing the color orange – statements beginning with “*I remember* …” relate these distinct subjective experiences, which, akin to feelings or sensations, are of different quality than mental states evoked when expressing other learned behaviors, such as habits, motor skills, or classical conditioning (see Box 1). Such private experiences of remembering are commonly referred to as qualia. Qualia may be an exclusive feature of the human mind, and we may never know to what extent we share them with the minds of other animals. But, as is the case with qualia, we all *know* what it is *like* to remember, thanks to introspection, and psychologists are often referring to this awareness when they use the term memory. This case can be most readily made for episodic memories, i.e., memories of events accompanied by the autonoetic awareness of remembering, but it is less clear for semantic memories, i.e., memories for facts. Memories, as qualia, do not exist as material things but are evoked mental states, and, although they appear to somehow depend on and correlate with brain functions, nowhere will they be found in the brain because they themselves are immaterial. Because qualia are immaterial, private, and subjective, “*memory*” in this sense cannot be addressed with reductionist methods.

**Box 1. A little glossary of commonly used terms in memory research.**

*Episodic Memory.* Memory for events, embedded into a remembered spatio-temporal context, necessarily accompanied by the awareness of remembering (autonoetic awareness), and thus, in the strictest possible sense, posited as exclusive to the human mind. Assumed to permit “mental time travel” because their remembering induces a feeling of vivid re-experiencing. Related to explicit memories. Probably largely reconstructed, not read-out.

*Semantic Memory.* Memory for factual knowledge akin to encyclopedic entries but also including such knowledge that things dropped will fall towards the floor and not float away into outer space. Some knowledge may be innate, or, with Kant, *a priori*, such as the intuitions of space and time, which guide development of acquired knowledge. Episodic memories make use of this factual knowledge, and are, in effect, populated with it. Thus, facts do not need to be remembered with the awareness of remembering, but they *can*, as is often the case during thinking with the awareness of thinking.

*Procedural Memory.* Learned motor skills, such as walking, riding a bike, putting on clothes, tying knots etc. Normally recalled without accompanying awareness that memories are being used, and performance usually decreases when trying to become aware of recalling while doing.

*Explicit vs. Implicit Memory.* Explicit memory shares awareness of recall with episodic memory; implicit memory, on the other hand, may be described as expressing memory without accompanying awareness that memory is being expressed, as is often the case with procedural memories for example. Thus, explicit memories are associated with distinctive private mental states, i.e., qualia, while implicit memories are not. Because they are qualia, we do not know whether non-human animals experience explicit memories.

*Classical Conditioning.* The term does not refer to memory but to the procedure of how a certain memory is acquired. In essence, classical conditioning is about knowledge of a prediction in the form of “if A, then B will follow (soon).” The acquired prediction could be classified as a form of semantic memory, but formally it is regarded as an association between a stimulus and a response, and stimulus as well as association are forms of memory.

*Long-Term Potentiation; Long-Term Facilitation.* A long-lasting increase in the strength of synaptic connectivity, measured in the magnitude of evoked post-synaptic response, as a result of either an experience that leads to behavioral changes or an artificial stimulus, usually designed to mimic the memory forming experience.

*Long-Term Depression.* A long-lasting decrease in the strength of synaptic connectivity, measured in the magnitude of evoked post-synaptic response, as a result of either an experience that leads to behavioral changes or an artificial stimulus, usually designed to mimic forgetting.

Neuroscientists also use the term “*memory*,” but usually not in referring to a quale, yet often without realizing it or an awareness that other scientists in the field do so. This generates confusions because the revelations of the reductionist methods used in neuroscience cannot address “*memory*” as defined by many psychologists. “*Memory*” addressed within neuroscience explores a fundamental property of many life forms, namely that experiences can alter future behavior, which can be studied in a variety of learned behaviors, such as habits, motor skills, or classical conditioning, without getting entangled with qualia. The question about memory then becomes a question about how biological processes allow these behaviors to emerge. Yet, inferences about the *experience* of remembering derived from the outcomes of these reductionist research efforts, which usually use behavior as proxy for memory, are mere surmise, and thus we should be mindful of how we talk about the generated data. Therefore, we should attempt to use terminology that accurately reflects what reductionist methods allow us to talk about, mindful whereof we must be silent.

## The Spell of the Engram

The inaccuracies arising from terms that blur the line between memory as qualia and memory as matter surround the recent revival of the notion of the engram (Box 2). Richard Semon introduced the term, proposing that sensory stimulation (German *Reiz*) leads to a permanent morphological change of the “excitable substance” (German *erregbare Substanz*) of the organism, i.e., in the case of animals, the nervous system (Semon suggested his theory applies to plants as well). Semon explicitly stated that he thought of a process akin to engraving, or carving (“I refer to the effect of sensory stimulation as their engraphic effect because they carve or inscribe themselves into the organic matter, so to speak.” German “*Ich bezeichne diese Wirkung der Reize als ihre engraphische Wirkung, weil sie sich in die organische Substanz sozusagen eingräbt oder einschreibt.*”; Semon, 1904). These *structural* (i.e., material) changes arising as a response to a particular stimulation Semon called the engram, i.e., an inscription (Greek *gramma*) placed into (Greek *en*) the nervous system. The sum total of all engrams are the mnemes of the organism. The idea captured in the notion of engram is thus not much different from the wax tablet analogy for memory Socrates develops in dialogue with Theaetetus: “[…] when we wish to remember anything […] we hold the wax to the perceptions and thoughts, and in that material receive the impression of them as from the seal of a ring; and that we remember and know what is imprinted as long as the image lasts; but when the image is effaced, or cannot be taken, then we forget and do not know.” Thus, the ideas of engram and wax tablet contain the notion of analog correspondence, in that engram corresponds to memory of the sensory stimulus, insinuating the ability to locate and control memories. Consequently, the idea of engram becomes attractive to modern positivist, reductionist researchers, encouraging dreams of “*engram technology*” and “*engram editing*” endowing us with the ability to directly control memories, changing, deleting, and evoking them at the press of a button (Ryan et al., 2015; Tonegawa et al., 2015), while in fact these methods have merely demonstrated they can control behavior.

**Box 2. Terminological alternatives.**

*Engram.* We suggest avoiding this term altogether. Instead, relatively more neutral terms like “cell assembly (supporting memory),” or “neuronal ensemble (supporting memory)” are less biasing when referring to changes in the brain that were caused by sensory experience and that are posited biological underpinnings of memory.

*Consolidation and Reconsolidation.* Instead of invoking the idea of consolidation it seems more parsimonious and less misleading to talk about the formation, construction, or generation of long-term memory that can resist a certain class of interferences at the behavioral and cellular level. This will not require additional assumptions about how short-term and long-term memories are linked. The same applies to the notion of reconsolidation, referring to a process that, similar to consolidation, converts unstable cellular states into stable ones. Here, the cleanest way to refer to the phenomenon might be reactivation-induced plasticity.

*Systems Consolidation.* This misnomer refers not to a stabilization, but a reorganizational, or transformational process that can be evoked for many long-term memories under various conditions, often involving retrieval, and seems to occur during sleep. It seems not possible to assign a firm timeline to “systems consolidation,” and some even have suggested it may take years in humans to complete, which in a sense questions its utility as a concept in the first place. It might therefore be more appropriate to refer to this continuous processing of memories as systems-level memory transformation, or curation, which may better reflect the idea that the majority of memories are updated and modified in various ways for as long as they are available.

While there is nothing wrong with the idea that sensory stimulation causes changes in brain morphology, the relationship between these changes and re-experiencing aspects of what initially gave rise to them will remain entirely unclear, as we have discussed above, most likely until these methods are used with humans. This issue is further complicated by the presumed relationship between overt behavior and memory – the fact that artificial brain stimulation causes a behavioral reaction similar to what can be observed when the organism makes natural use of acquired knowledge does not mean that in both cases memory or the same kind of memory is at play, although it is tempting to tacitly assume so (Hardt and Nadel, 2017). This issue touches the core of the hard problem of consciousness, i.e., the relationship between brain activities and qualia, or private experiences (Grossberg, 2017). This question remains (possibly forever) unanswered and reminds us to be careful using language that reflects absence of knowledge when talking about behavior, brain processes (engram), and subjective experiences when remembering (quale).

For example, the latest advances in neuroscience endowed us with the ability to label neurons that expressed an activity marker (*cFos*) during sensory stimulation and then deliver only to these neurons mRNA to express specialized membrane channels sensitive to a certain light wavelength. This allows polarizing or depolarizing the infected neurons to inhibit or excite only the very neurons that once were active when the brain reacted to the original stimulation. Using this method in the hippocampus during contextual fear conditioning, animals will thus freeze whenever the labeled neurons are fired at a certain frequency, and it has been suggested that these neurons that, when fired, cause animals to freeze, form an “*engram*” (Nakashiba et al., 2009).

Although, in hindsight, it is not surprising that reactivating the very neurons that once were active when a behavior was observed makes animals express similar behavior, it is not clear what this outcome really means. First, there is this problematic notion of the stability of cell assemblies associated with memory. The vast majority of optogenetic studies exploring memory target the hippocampus, where neural populations involved in a particular spatial memory change over time, overlapping only around 15% when memories are tracked for 30 days (Ziv et al., 2013). Similarly, in the hippocampus, dendritic spines do not persist for very long, disappearing within 7–14 days (Attardo et al., 2015), suggesting that either memories are normally lost from the hippocampus within this timeframe (Hardt et al., 2013; Migues et al., 2016) or that the neurobiological “substrate” of memory in the hippocampus is indeed highly dynamic and regularly reallocated to different sets of neurons. The remapping phenomenon is another indicator that populations of neurons recruited to represent memory are not stable over time, and that, in fact, a set of neurons can participate in a multitude of memories, which seems not what one would expect from the connotations surrounding the term engram, the conceptualizations of memory it encourages, and the experimental designs it inspires. Indeed, while all of the neurons that had expressed *cFos* (or any other activity marker) during learning are reactivated in these optogenetic experiments, a much smaller percentage are reactivated during natural recall (Reijmers et al., 2007; Tayler et al., 2013). Thus, it is quite unclear whether the brain would recruit the same neurons when animals naturally express the behavior elicited in these studies. Second, it is unresolved whether for a certain memory entire neurons are recruited, or rather a selected group of synapses, yet calling these the “*engram*” cells insinuates this issue is settled and that the fundamental unit of the presumed biological substrate of memories is the individual neuron itself. Third, the pattern of activity induced by optogenetics is quite different from the activity natural stimuli induce (e.g., place fields firing in sequence when an animal explores a context are fired all at once in optogenetic stimulation), raising the question whether and how the expressed behavior relates to what animals experience: does optogenetically (or otherwise) induced “artificial” freezing mean the animal experiences fear or does it simply just freeze? To conclude, the actual status of the optogenetically targeted neurons in relationship to memory remains thoroughly undefined. Yet, this crucial issue is at the very heart of the actual problem these research efforts seem to target, or at least wish to address.

Apart from these epistemological confusions, there are profound conceptual consequences of using the word “*engram*” – its very notion of *engraving*, *inscribing* or *imprinting* incorporates the idea that relatively static structures underpin memories, which hardly fade and whither over time, remain generally unpliable, require significant effort to be modified, and largely stay fixed in form and place. On the one hand, this position encourages expeditions in search of “*the engram*,” with the ultimate aim to locate the seat of memories in the brain (Lashley, 1950). It is unclear whether these renewed attempts to find the engram will face a similar fate as Lashley’s original quest, arriving eventually at the same conclusion that constituents of memories are distributed all over the brain, i.e., memories are everywhere and nowhere. On the other hand, the idea of stasis clearly mischaracterizes the fundamentally dynamic nature of memory, reflected both in its underpinning neurobiology and in the plethora of cognitive phenomena demonstrating that what we call “remembering” results from elaborate regenerating and reconstructing on the background of substantial forgetting and distortions, and not from reading out an engraved epitaph, as the term engram might insinuate (Hardt et al., 2010, 2013; Barry and Maguire, 2018). Stated succinctly, the term engram may reflect more wishful thinking than how memory and brain actually relate (Box 2).

## Panta Rhei

Wherever you look, memory behaves inherently like a fluid, not a solid. On the cognitive-behavioral level, phenomena like hindsight bias, misinformation effect, context-dependent updating, hypermnesia, reminiscence, etc. demonstrate that the act of remembering can irrevocably alter the contents of established long-term memories, while various forms of forgetting suggest that the content of dormant long-term memories that is deemed irrelevant, superfluous, or limiting may be erased or suppressed (Hardt et al., 2010). On the cellular level, the proteins implementing the molecular alterations underpinning cell assemblies that are somehow involved in memories are in a constant state of flux, with proteins being turned over, translocated, reconfigured, substituted, and replaced (Baltaci et al., 2019). Yet, the general perception of memory, and the words used to describe it, suggest a static system characterized by the goal of preserving records of past experiences with high fidelity, in contrast to the reality of an inherently adaptive system purposed to enable survival in a changing world with a pragmatic disregard for the fate of acquired memories that are outdated or otherwise inadequate. Indeed, the idea that accurate prediction of what might likely happen given certain conditions may constitute the main purpose of brain and mind increasingly gains attention and support. Yet, the fundamental concepts in memory research suggest otherwise.

Once declared the dogma of the century (Mcgaugh, 2000), the idea of memory consolidation (Box 2) arose from the finding that sensory stimulation affects recent human memories more so than remote ones (Müller and Pilzecker, 1900). Thus, it was assumed that memories progressively consolidate, becoming increasingly more resistant to various forms of interference, an idea that is still strong today. Consolidation presently has two meanings, which refer to completely different types of processes – either to *synaptic* changes (cellular consolidation) or brain *systems* dynamics (systems consolidation) associated with memory formation. To complicate things further, students of human memory traditionally use the term consolidation without either qualifier (i.e., without specifying whether they address synaptic or systems consolidation), referring more to a cognitive process that somehow stabilizes newly acquired memories.

### Cellular Consolidation

This term arose from the observation that long-lasting changes in synaptic strength are sensitive to disruptors (e.g., protein-synthesis inhibitors, seizures) only for a short period during and after learning (Mcgaugh, 2000). It was first shown using electroconvulsive seizures in rats (Duncan, 1949) and protein-synthesis inhibitors in the goldfish (Agranoff et al., 1965). When these kinds of treatments no longer affect synaptic changes or overt behavior, consolidation is said to have completed. Consolidation thus embodies the idea of converting an unstable form (short-term memory) into a stable one (long-term memory). This link is troublesome for two main reasons.

First, many models for how long-lasting changes in synaptic strength are achieved lack a well-developed mechanism converting unstable, short-lasting changes in synaptic strength to stable, long-lasting ones. For example, leading models propose that the number of synapses within neuronal assemblies *increases* during cellular consolidation (Bailey et al., 2015; Tonegawa et al., 2015; Choi et al., 2018), and that learning involves both *generating* and stabilizing new synapses as well as *pruning* others (Xu et al., 2009). The processes ‘generating’, ‘forming’, or ‘constructing’ (Box 2) these *long-lasting* changes to synaptic connectivity, however, cannot be *identical* to those supporting *short-lasting* changes because otherwise the latter would suffice to form long-term memory. This issue often overlooked has not been resolved. Alternatively, a set of different processes that all start at the same time could implement short-lasting and long-lasting increases in connectivity so that converting the former into the latter will not be necessary because they each are supported by relatively independent mechanisms (Izquierdo et al., 2002; Sossin, 2008). For example, increasing the number of excitatory receptors and maintaining these with a dedicated process could enhance short-lasting connectivity (Lisman, 2003, 2017); sprouting new functional synapses, possibly requiring additional signaling events, such as the release of neuromodulators, could promote long-lasting increases in connectivity (Sajikumar and Frey, 2004; Reymann and Frey, 2007). Furthermore, behavioral differences distinguish short- and long-term memories (Cammarota et al., 2007; Cowan, 2008). This parsimonious two-component model can explain why certain forms of interference differentially affect short-lasting and long-lasting changes to connectivity.

The second problem associated with the concept of consolidation relates to it being incongruous with several phenomena suggesting that the cellular components related to memory are anything but stable. For example, the assumed “*consolidated*” and “*stable*” morphological changes to synapses nevertheless require *constant* care and maintenance. A continuously active mechanism controls the ongoing trafficking of GluA2-containing AMPA receptors at synapses involved in long-term memory, regulating the persistent movement of the very proteins implementing the so-called “*consolidated*” increases in connectivity (Migues et al., 2010; Dong et al., 2015). This process maintains itself with an elegant combination of feedforward loops, illustrating that apparent stability can arise from actual incessant flux of the constituent components. Then, there are active forgetting (Hardt et al., 2013; Davis and Zhong, 2017) and retrieval (Hardt et al., 2010) that substantially alter the presumably consolidated changes in synaptic strength of long-term memory representations in a process of reactivation-induced plasticity (Box 2) (Nader and Hardt, 2009). Thus, dormant or in use, the neurobiological “*substrate*” of memory resembles a fluid more than a solid, always ready to “morph” into new configurations, and yet long-lasting memories are possible despite the constant turnovers and fluctuations of this presumed substrate (Trettenbrein, 2016). This primary quality, the fundamentally *plasmic* nature of the “memory substrate,” is not at all reflected in terms like engram or consolidation (Box 2).

The finding that specific neuronal ensembles can be associated with memory has distracted attention away from synapses to neurons to understand memory. However, this alternative view ignores the problem of exorbitant loss in computational power if memories were encoded at the cellular instead of the synaptic level. Moreover, models proposing that some form of nuclear codes store learning-induced changes in synaptic connectivity lack mechanisms explaining substantiation, which is not surprising considering that there is no conceivable model to solve the substantiation problem (Sossin, 2018). In this context, it may be useful to address how we talk about the actual synapses that encode the changes in synaptic strength that may underlie memory. There is robust evidence supporting the idea that there are synapses specialized in encoding long-term changes in synaptic strength. The evidence supporting these “memory synapses” (Sossin, 2018) mainly consists of studies in which specifically the increases in synaptic strength induced by learning are erased, leading to the loss of memory without affecting the majority of synapses present (reviewed in Sossin, 2018). However, in keeping with the overall goal to develop nomenclature that is more precise and less leading, and since the evidence for this specialization is their ability to be specifically erased, we suggest calling these particular synapses “synapses vulnerable to erasure (SVEs)” because they are the ones that are maintained, lost, and modified during long-term memory formation, retention, retrieval, and forgetting.

#### Ways Forward

What have we actually learned about cellular processes involved in the generation of long-term memories? Long-term potentiation (LTP) used to be a good reference point shortly after the phenomenon was discovered. Originally, LTP referred to a long-lasting *increase in the synaptic response (potentiation)* resulting from stimulation at high frequency (Bliss and Lomo, 1973). Over the years this term became fuzzy as it has been applied to pretty much any increase in synaptic strength regardless of the specific induction procedure. In invertebrates, increases in synaptic strength have usually been termed facilitation, where the word originated in the pioneering work of Eccles (1935). Facilitation now is used in invertebrates even when stimulating at high frequency (Hu and Schacher, 2015), with maintenance mechanisms likely similar to LTP (Hu et al., 2017). If any and all forms of stimulation-induced increases in synaptic strength are called LTP, then this debate in fact is only about how long-term increases in synaptic strength relate to memory, not about what LTP specifically can tell us about the molecular mechanisms underpinning memory. Indeed, Malenka and Bear (2004) remind us that “given the ubiquity of various forms of LTP and LTD at excitatory synapses throughout the brain and the clear computational advantages they afford, it seems virtually certain that the brain takes advantage of the neuronal capability to express long-lasting activity-dependent synaptic modifications as at least one of the key mechanisms by which experiences modify neural circuit behavior.”

Terminological precision is important for the continuous discussion concerning the relationship between LTP and memory. Molecular or pharmacological strategies that block LTP but do not disrupt memory are easily dismissed by the assertion that a distinct form of LTP encodes the memory. Similarly, correlations between molecular mechanism underlying a form of LTP and a type of memory can be dismissed if the induction mechanism for that particular form of LTP does not match the firing patters seen *in vivo* (see Takeuchi et al., 2014, for a detailed discussion of these arguments). Moreover, sophisticated homeostatic mechanisms can compensate for a loss of a specific form of plasticity (El-Brolosy and Stainier, 2017; Turrigiano, 2017). This limits the conclusions that can be drawn from the outcomes of interfering with a certain cellular or molecular process: eliminating a particular mechanism may not eliminate the ability of an organism to make a memory, even though under normal conditions the mechanism contributes (Tsokas et al., 2016).

We therefore suggest focusing our inquiries into the neurobiological underpinnings of memory on the actual molecular mechanisms underlying increases in synaptic strength, asking more meaningful questions, such as: Are increases in AMPA receptor numbers involved in memory? What about increases in persistent protein kinase activity? Increase in the probability of presynaptic glutamate release? Connectivity increases? The question about the neurobiological processes critical for memory thus should address which *molecular/biochemical/structural mechanisms* are involved in memory and whether one can link these kinds of changes seen in a reduced system to what occurs in an intact living system (Sossin, 2008).

## Systems Consolidation

Among the terms discussed so far, systems consolidation may be the most confusing. It relates to a phenomenon first observed in patients with lesions to the hippocampus, notably H.M., suggesting that episodic memories, or memories of events, initially require the hippocampus, but after some time no longer seem to do so (Nadel and Hardt, 2011). It appears that over time areas outside hippocampus, such as regions of the frontal lobe, become progressively more involved in expressing these types of memories. It should be noted here that the idea of systems consolidation does not rest on the notion that information from the hippocampus is transferred out of hippocampus to some other place. Instead, since encoding, a set of neuronal ensembles, distributed throughout various brain regions, is involved in these memories, each representing particular aspects of the experience. What seems to change over time is the relative contributions of these various regions to a memory. The possible consequences of this for content and quality of memory have been debated for decades, and remain to be resolved (Hardt and Nadel, 2017).

Similar to the phenomenon captured in the term cellular consolidation, systems consolidation refers to an increasing insensitivity of a memory to certain disruptors, in form of various types of interference with hippocampal function, such as, for example, lesion or inactivation. It is misleading to classify this as a stabilization, or consolidation because the ability of a memory to resist a particular interference, does not imply it is more stable than before. Ignoring for a moment the lively debate about whether indeed true episodic memories, recent or remote, can do without the hippocampus, memories are not more stable because the hippocampus seems less important for expressing them. The fact that retrieving a “*systems consolidated*” memory can reengage the hippocampus (“*systems reconsolidation*,” Einarsson et al., 2014) further supports the view that the term is fundamentally misleading. Instead, as is the case for “*cellular consolidation*,” these findings suggest that mnemonic processes operating on the systems level are as dynamic as those supporting memory representations on the cellular level (Barry and Maguire, 2018).

What these findings do suggest, however, is that neuronal ensembles supporting event memories are continuously restructured in a perpetual transformational process (Box 2) (Dudai, 2012). There is no need to define a terminal state that this process needs to reach. Instead, we take the perspective that the very nature of memory, namely enabling and promoting adaptive behavior by improving predictions based on available knowledge, is incompatible with teleological notions of unidirectional mechanisms defined by stable, finite endpoints. Notwithstanding exceptionally stable memories (such as knowledge acquired during imprinting), the vast majority of memories are regularly refined and reprocessed to keep them current and to maintain a coherent model of the known world. The human as well as animal literature contains numerous cognitive phenomena illustrating this basic point (to name a few, misinformation effect, hindsight bias, schematization, Hardt et al., 2010), and several mechanisms have been suggested as being relevant for this, such as transient reactivation-induced plasticity or hippocampal replay.

## Final Thoughts and a Justification

The current situation in memory research reminds us of the time when Newtonian mechanics met quantum physics. Thus, from a macroscopic perspective, memory might indeed seem like a wax tablet and talking about it in such terms makes sense but looking at the microscopic level reveals that such analogies betray the nature of its actual underpinnings. Hence our aim to provoke a debate about terminology and concepts in the field of memory research. We believe it is time to carefully reconsider traditional terms that once had a well-defined meaning, but over the years became vague and almost prosaic, attached to a variety of possible interpretations, insinuations and connotations (Box 2). Additionally, it is often unclear whether there is a relation between qualia (which cannot be observed) and presumably relevant neurobiological processes (which can be observed). This issue gets potentiated when vague terminology meets high-precision technology because the outcomes of these advanced methods are taken as highly reliable confirmations of actually vague ideas and concepts. Using precise and perhaps more technical language when talking about neurobiological phenomena related to memory represents not a mere matter of style but an epistemological virtue.

Concepts guide thinking and influence scientific explorations during which we tend to recognize what we already believe to know. From a linguistic perspective our suggestions for more accurate terms may seem crude and unwieldy, but promoting a relatively unbiased, unprejudiced, detached and innocent scientific exploration with precise terminology will allow us to step outside our traditions and customary habits of thought, making it easier for us to take alternative perspectives due to a better awareness of what we actually know and potentially *can* know. A language that tries to keep close to the observables supports this sort of unconditional approach to the neurobiology of memory.

## Author Contributions

Both authors contributed equally to this perspective.

## Funding

OH is supported by CIHR (133444) and the Simons Initiative for the Developing Brain. WS is supported by CIHR (340328).
